# Corneal ectasia following cataract extraction surgery in a patient with keratoconus: a case report

**DOI:** 10.1186/s13256-019-2238-x

**Published:** 2019-09-19

**Authors:** Georgios Labiris, Eirini-Kanella Panagiotopoulou, Panagiota Ntonti, Sergios Taliantzis

**Affiliations:** Department of Ophthalmology, University Hospital of Alexandroupolis, Dragana, 68100 Alexandroupolis, Greece

**Keywords:** Keratoconus reactivation, Phacoemulsification surgery, Two-hit hypothesis

## Abstract

**Background:**

According to experimental and clinical published studies, patients with keratoconus have a genetic predisposition to corneal ectasia; however, ectasia might not be activated or reactivated unless an additional stressful event triggers the disease. Triggering factors are sources of reactive oxidative stress; among them, mechanical trauma (vigorous eye rubbing, poorly fit contact lenses), exposure to ultraviolet light, and atopy/allergies. The aim of this case report is to present for the first time a case of rapidly progressive corneal ectasia in a patient with keratoconus following uncomplicated phacoemulsification surgery for cataract removal.

**Case presentation:**

A 38-year-old Caucasian man was referred to our out-patient’s service due to bilateral cataract. He also had bilateral keratoconus and had undergone corneal cross-linking in both his eyes 5 years prior to his referral. Ever since the corneal cross-linking, keratoconus had been stable. He underwent a full ophthalmological examination including slit-lamp biomicroscopy, optical biometry, Scheimpflug tomography, corneal biomechanical assessment, and fundus examination. He presented advanced centrally located cataract with count fingers for preoperative best-corrected visual acuity. An uncomplicated cataract extraction surgery was performed. Preoperative flat keratometry reading was 40.5 diopters, steep keratometry reading was 41.8 diopters, astigmatism was 1.3 diopters, corneal hysteresis was 8.2, corneal resistance factor was 7.5, and thinnest corneal thickness was 503 μm. Within 3 months, he demonstrated rapidly progressing corneal ectasia in his operated eye, while 6 months postoperatively, flat keratometry reading was 45.5 diopters, steep keratometry reading was 48.3 diopters, astigmatism was 2.8 diopters, corneal hysteresis = 6.8, corneal resistance factor = 7.5, and thinnest corneal thickness = 318 μm.

**Conclusions:**

To the best of our knowledge, this is the first report to describe corneal ectasia in a patient with keratoconus following phacoemulsification surgery. Cataract surgeons should provide extra caution to patients with keratoconus and take into consideration this rare but potentially sight-threatening complication.

## Background

It is known that keratoconus (KC) is a slowly progressive, non-inflammatory disorder characterized by thinning of the inferior or central stroma and anterior corneal protrusion. Corneal thinning results in corneal architectural distortion, irregular astigmatism, myopia, and significant corneal aberrations [1].

Corneal cross-linking (CXL) is considered the only therapeutic technique that attempts to interrupt the natural progression of the disease and not simply address the refractive error. CXL uses riboflavin and ultraviolet A radiation. Either the traditional Dresden protocol or the contemporary faster protocols stabilize the collagen matrix in corneas with KC and stop or minimize further ectasia. It may be combined with photorefractive keratectomy (PRK) in order to improve astigmatism and other higher order aberrations. In particular, combined PRK-CXL treatments seem to improve significantly the quality of life of patients with KC [2].

Regardless of KC treatment approaches, the majority of eyes with KC present limited or no progression after the age of 30 due to natural cross-linking-like alterations of the cornea by sunlight. However, certain eyes with KC do progress; although the exact pathomechanism is yet to be explored [3].

It is not unusual that certain treatment options, pharmaceutical or surgical, might initiate or accelerate the progression of KC. A recent publication presented a case of rapid progression of KC in a 49-year-old woman on selective tissue estrogenic activity regulator therapy for endometriosis [4]. The authors suggested that women under this specific treatment should be closely monitored for corneal changes. Within this context, we would like to present a case of rapid progressive corneal ectasia in a patient with KC following conventional phacoemulsification surgery for cataract removal. To the best of our knowledge, this is the first report to describe KC reactivation following phacoemulsification surgery.

## Case presentation

A 38-year-old Caucasian man was referred to our out-patient’s service from his physician due to bilateral cataract. His systemic medical and family history was negative. He was receiving no medication; he did not smoke tobacco or consume alcohol; he worked as a clerk in a bank. His ophthalmological history indicated bilateral KC with myopic astigmatism, for which he received CXL (Dresden protocol) in both his eyes 5 years prior to his visit to our hospital. According to his medical note, ever since the CXL, KC had been stable.

**Fig. 1:**
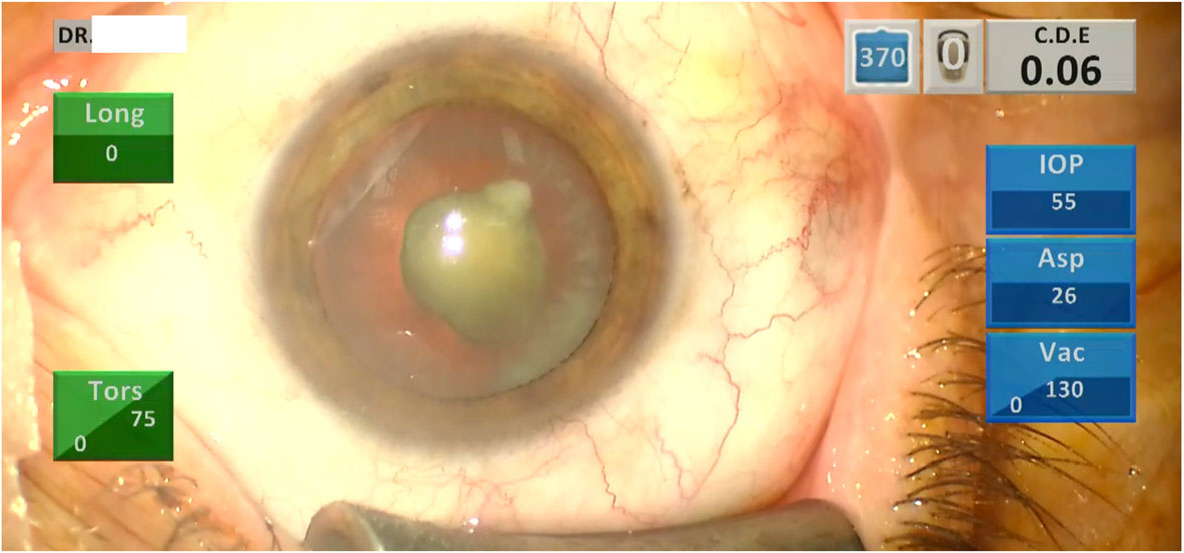
Image of the cataract prior to phacoemulsification surgery from the surgical microscope’s camera

**Fig. 2:**
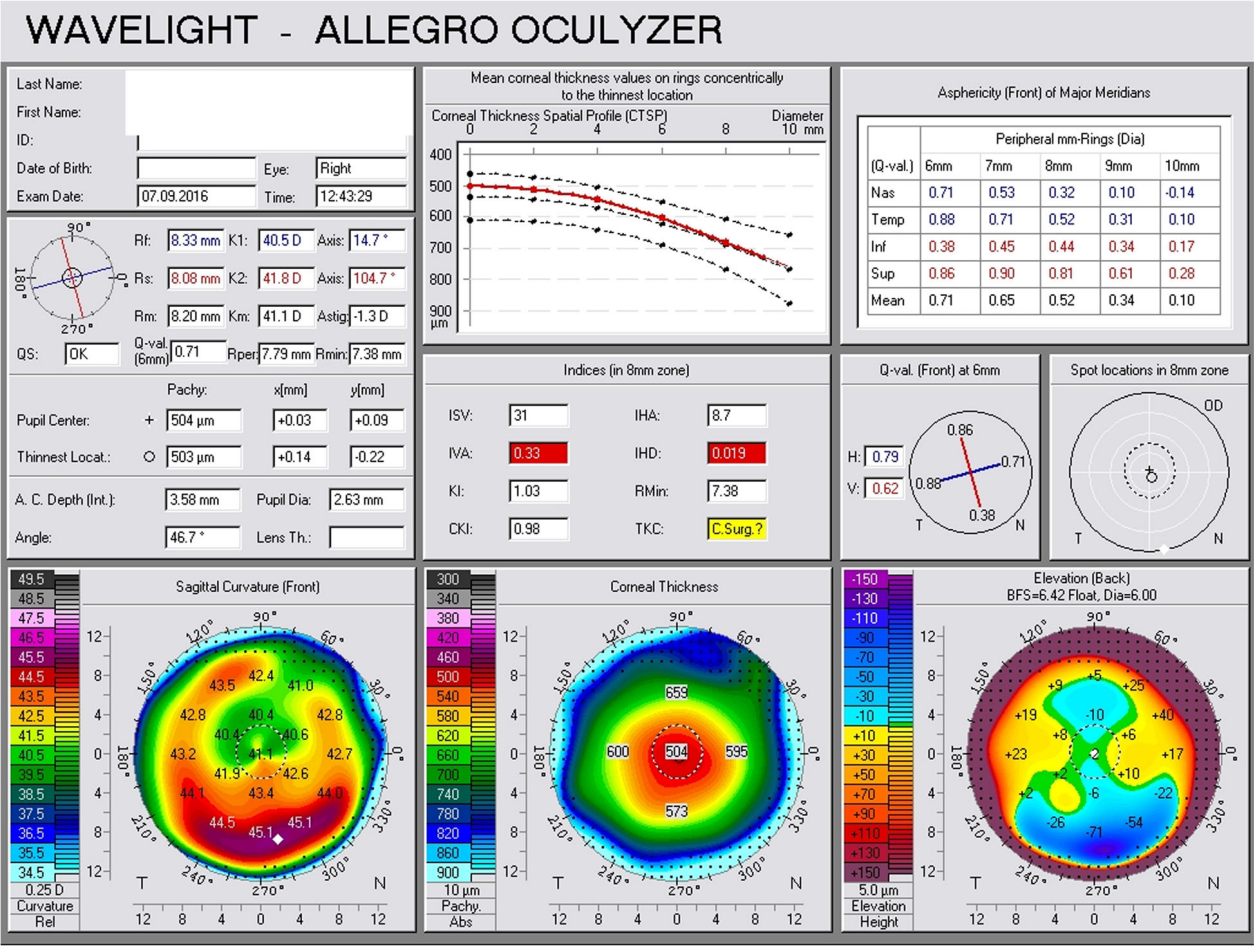
Scheimpflug tomography of the right eye prior to phacoemulsification surgery

**Fig. 3:**
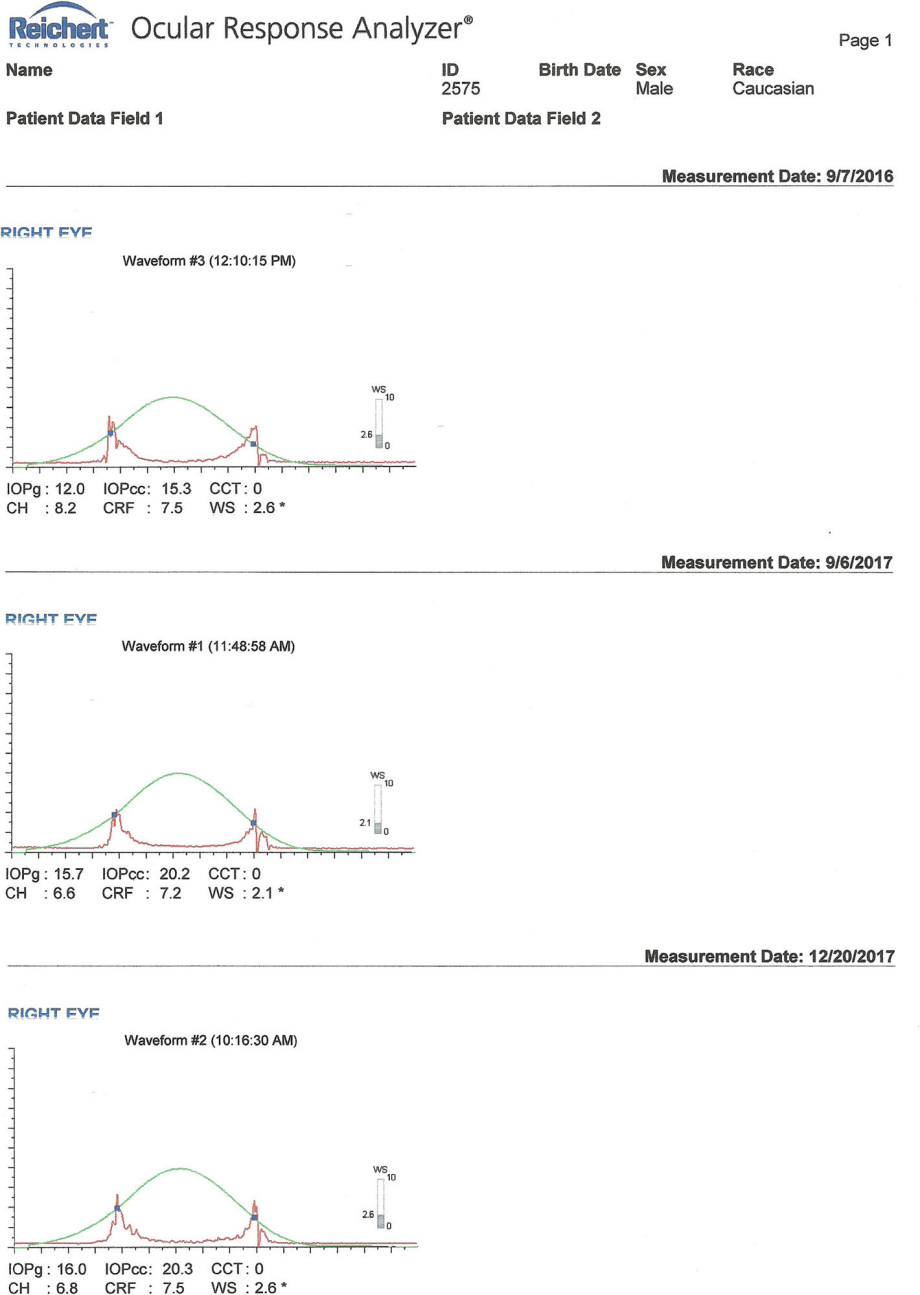
Waveform #3: ocular response analyzer measurements of the right eye prior to phacoemulsification surgery. Waveform #1: ocular response analyzer measurements of the right eye 3 months postoperatively. Waveform #2: ocular response analyzer measurements of the right eye 6 months postoperatively

He underwent a thorough systemic examination that did not reveal any pathological signs. On admission, his heart rate was 70 beats per minute, his blood pressure was 120/70 mmHg, and he had a normal body temperature. Subsequently, he underwent a full ophthalmological examination including slit-lamp biomicroscopy, optical biometry, Scheimpflug tomography, corneal biomechanical assessment, and fundus examination. During the examination we confirmed advanced centrally located cataract (Fig. 1) and: (a) index of height decentration (IHD) = 0.019; (b) index of vertical asymmetry (IVA) = 0.33, which are common signs of post-CXL corneas (Fig. 2). His best spectacle-corrected visual acuity (BSCVA) was count fingers (CF) for both eyes. Preoperative flat keratometry (K1) reading was 40.5 diopters (D), steep keratometry (K2) reading was 41.8 D, astigmatism was 1.3 D, and thinnest corneal thickness (TCT) was 503 μm (Fig. 2). His corneal hysteresis (CH) was 8.2 while his corneal resistance factor (CRF) was 7.5, which were consistent with KC and post-CXL corneas (Fig. 3, waveform #3) [5, 6]. His fellow eye demonstrated K1 = 40.9 D, K2 = 41.9 D, IHD = 0.041, IVA = 0.67, CH = 8.0, and CRF = 7.3. Fundus examination turned negative for both eyes.

We decided to propose phacoemulsification surgery for his right eye with intraocular lens (IOL) implantation. Our patient consented to the operation and we proceeded to an uncomplicated phacoemulsification with Alcon Infiniti® Vision System platform (80% continuous amplitude with 350 mmHg vacuum limit and 40 mL/minute aspiration flow rate) with 2.2 clear-corneal main incision and two contralateral stabs. A foldable hydrophilic acrylic IOL (SN60WF, Alcon Laboratories, Inc.) was implanted, and our patient was released the same day with fixed combination of tobramycin 0.3% and dexamethasone 0.1% (FCTD) (Tobradex; Alcon, Greece) six times daily, gradually tapered over a month. His uncorrected visual acuity (UVA) during the first week was improved to 20/32, BSCVA = 20/25, his intraocular pressure (IOP) was 17 mmHg, and slit-lamp biomicroscopy revealed minor endothelial striae that were attributed to the phacoemulsification energy.

**Fig. 4:**
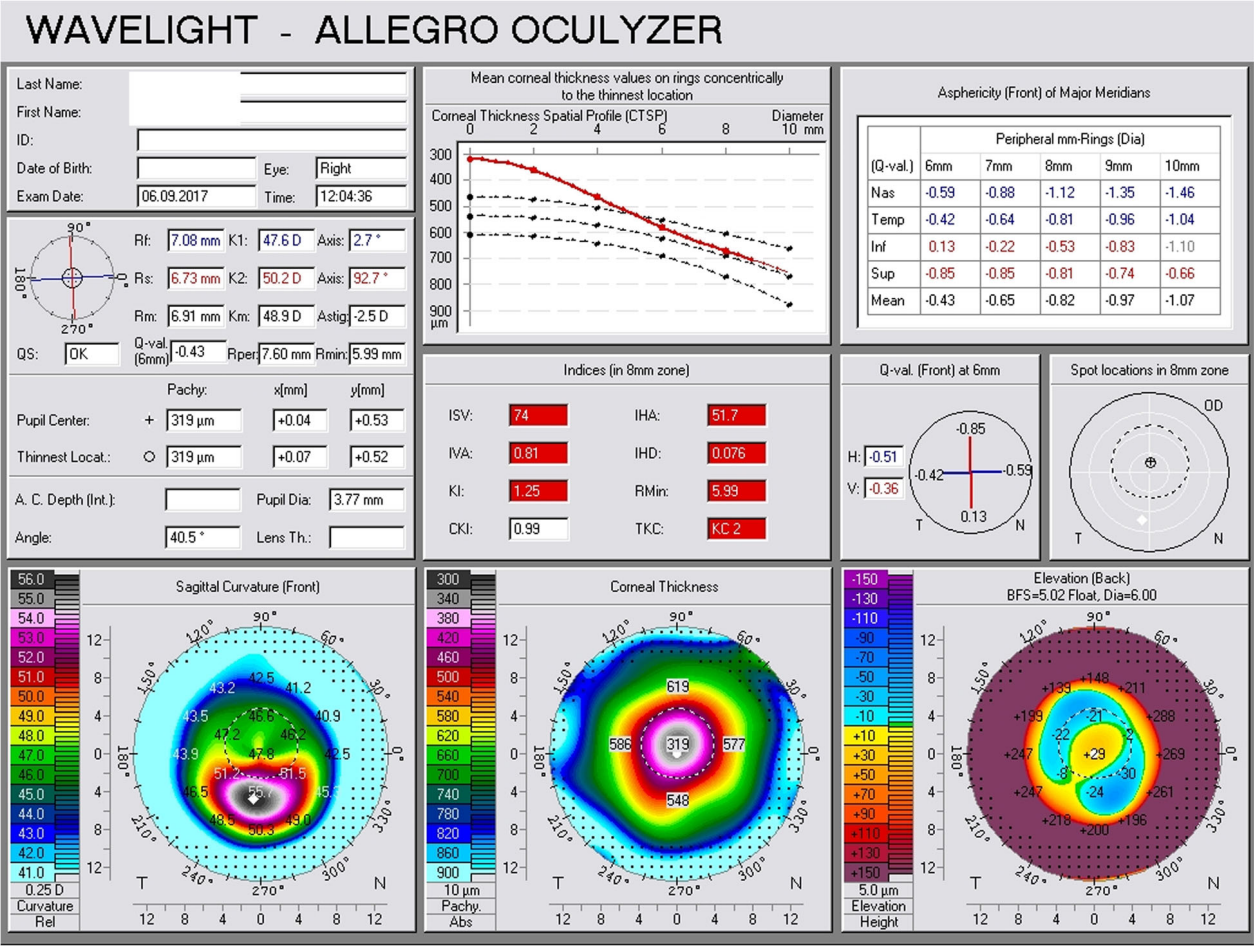
Scheimpflug tomography of the right eye 3 months postoperatively

**Fig. 5:**
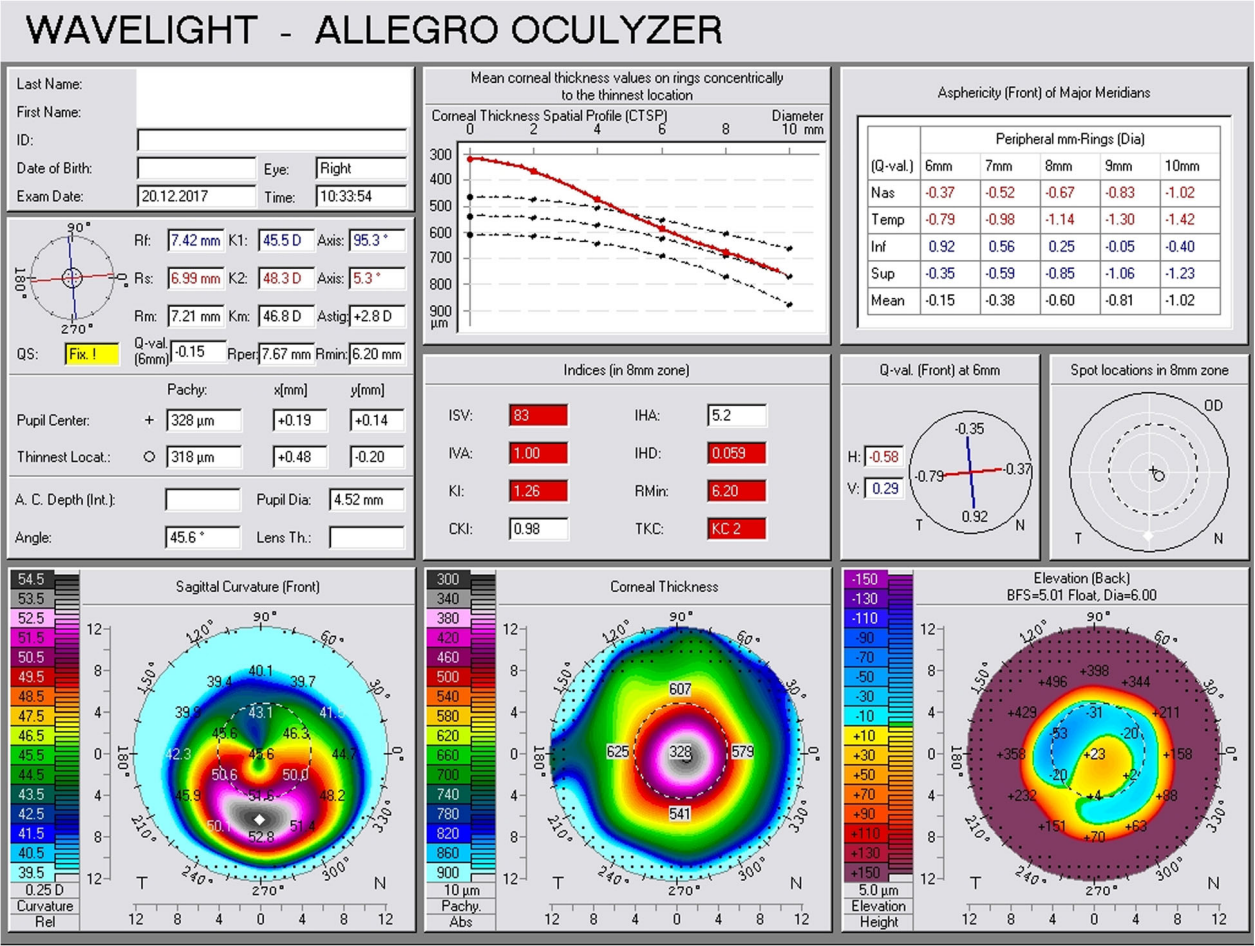
Scheimpflug tomography of the right eye 3 months postoperatively

Three months following the phacoemulsification, he was referred again to our hospital from his physician due to constantly increasing myopic astigmatism. We performed a Scheimpflug tomography and we detected significant corneal thinning, corneal protrusion (K1 = 47.6 D, K2 = 50.2 D), TCT = 319 μm (Fig. 4), and manifest refraction (− 5.00 sph − 2.50 cyl 180^o^). His UVA in his right eye was CF, while his BSCVA dropped to 20/32. All tomographical signs suggested potential corneal ectasia. To our surprise, ocular response analyzer evaluation confirmed a biomechanical destabilization of the cornea with significant reduction of the CH = 6.6 and CRF = 7.2, (Fig. 3, waveform #1). Six months following the cataract extraction surgery, both Scheimpflug tomography and ocular response analyzer demonstrated slightly improved tomographical and biomechanical indexes; however, they were fully indicative of postoperative ectasia (K1 = 45.5 D, K2 = 48.3 D), astigmatism = 2.8 D, CH = 6.8, CRF = 7.5, TCT = 318 μm (Figs. 3 and 5, waveform #2). His UVA in his right eye remained CF, while his BSCVA was 20/32. Non-significant changes in the K1 and K2 readings could be detected in his left eye (K1 = 40.8 D, K2 = 42.0 D) and in BSCVA which did not change and remained CF.

## Discussion and conclusions

The present report describes the appearance of corneal ectasia following cataract extraction surgery in a patient with KC, despite former CXL treatment. It is known that the exact etiology of KC remains unclear. However, scientific evidence has indicated that KC is a multifactorial, multigenic disorder involving complex interaction of not only genetic, but also environmental factors. According to experimental and clinical published studies, patients with KC have a genetic predisposition to corneal ectasia (first hit); however, ectasia might not be activated or reactivated unless an additional stressful event (second hit) triggers the disease (“two-hit” hypothesis). Triggering factors are sources of reactive oxidative stress; among them, mechanical trauma (vigorous eye rubbing, poorly fit contact lenses), exposure to ultraviolet light, and atopy/allergies [1, 7, 8].

Phacoemulsification surgery for cataract extraction is known: (a) to induce corneal mechanical stress due to corneal incisions, primarily during IOL implantation; and (b) to destabilize tear film and promote ocular surface disease. Possibly, in our patient, phacoemulsification-induced stress acted as the triggering factor that destabilized the cornea, despite the fact that our patient had already received CXL and was supposed to have minimal risk for ectasia. It should be mentioned that both tomographic and biomechanical indices of the fellow eye remained constant, suggesting that no systemic or environmental cause induced the ectatic phenomenon.

To the best of our knowledge, this is the first report to describe KC reactivation following phacoemulsification surgery. We do not know whether modification of the postoperative treatment, possibly with the use of additional non-steroidal anti-inflammatory drops, might have prevented the ectasia. Furthermore, a literature review returned no published reports on a potential contribution of FCTD to the ectatic phenomenon. However, cataract surgeons should provide extra caution to patients with KC and take into consideration this rare but potentially sight-threatening complication.

## Data Availability

De-identified data are available in print form for 1 year following the conclusion of the study.

## Abbreviations

BSCVA: Best spectacle-corrected visual acuity
CF: Count fingers
CH: Corneal hysteresis
CRF: Corneal resistance factor
CXL: Corneal cross-linking
D: Diopters
FCTD: Fixed combination of tobramycin 0.3% and dexamethasone 0.1%
IHD: Index of height decentration
IOL: Intraocular lens
IVA: Index of vertical asymmetry
K1: Flat keratometry
K2: Steep keratometry
KC: Keratoconus
PRK: Photorefractive keratectomy
TCT: Thinnest corneal thickness
UVA: Uncorrected visual acuity
